# Count Data Time Series Modelling in Julia—The CountTimeSeries.jl Package and Applications

**DOI:** 10.3390/e23060666

**Published:** 2021-05-25

**Authors:** Manuel Stapper

**Affiliations:** Institute of Econometrics and Economic Statistics, Westfälische Wilhelms-Universität Münster, 48149 Münster, Germany; manuel.stapper@wiwi.uni-muenster.de

**Keywords:** count data, time series analysis, Julia programming language

## Abstract

A new software package for the Julia language, CountTimeSeries.jl, is under review, which provides likelihood based methods for integer-valued time series. The package’s functionalities are showcased in a simulation study on finite sample properties of Maximum Likelihood (ML) estimation and three real-life data applications. First, the number of newly infected COVID-19 patients is predicted. Then, previous findings on the need for overdispersion and zero inflation are reviewed in an application on animal submissions in New Zealand. Further, information criteria are used for model selection to investigate patterns in corporate insolvencies in Rhineland-Palatinate. Theoretical background and implementation details are described, and complete code for all applications is provided online. The CountTimeSeries package is available at the general Julia package registry.

## 1. Introduction

The collection of count data dates back to at least 4000 years ago when the first census was documented. Nowadays, it still plays an important role in our everyday life as it occurs in various fields. For instance, it arises in insurance with the number of claims, in epidemiology where the number of infected patients is recorded, in urban planning as traffic volume, or in marketing with the number of certain items sold, just to name a few. During the past decades, researchers have developed methods to gain insight into the structure of the data and derive conclusions from it. Important frameworks for such analyses are integer counterparts of the well-known ARMA and GARCH models, the INARMA model introduced by Alzaid and Al-Osh [1], and the INGARCH model described, for example, by Ferland et al. [2]. Both models, the INARMA and the INGARCH model, have been further extended thereafter and are well established. In the following applications, a software package for the Julia Programming Language (Bezanzon et al. [3]) is used, which provides methods for these two models, CountTimeSeries.jl. After the Julia language was first published in 2012, the number of users grew remarkably during past years. On the one hand, it is as easy to learn as an interpreted language like R or Python, and on the other hand, it is a compiled language which makes its computation time comparable to C and Fortran. This combination makes Julia attractive for both researchers and practitioners. The CountTimeSeries package follows the idea of the language itself by providing a toolbox for count data time series that is simple to use and at the same time fast enough to allow analyzing large time series or carry out simulation studies. Covering some important generalization of INARMA and INGARCH models, the package allows to simulate from them, estimate parameters and conduct inference, assess the model choice, and compute forecasts.

There is no comparable package in R or Julia that covers the same functionalities. One important package for count data time series is R’s tscount package, see Liboschik et al. [4]. In contrast to the CountTimeSeries package, it provides residual assessment, calibration methods, and intervention analysis. However, it does not cover the INARMA framework at all. Further, it also does not incorporate zero inflation, a model property frequently used in count data analysis. The thinning-based INARMA model can be formulated as Hidden Markov model, as for example discussed by Weiß et al. [5], so that the R packages HiddenMarkov (Harte [6]), and HMM (Himmelmann [7]) can be used to estimate its parameters. Both packages have been developed for general HMMs, thus the CountTimeSeries package offers the advantage of a more convenient usage and similar notation for both frameworks making it easy to switch between frameworks or compare results between those. As the name already suggests, count regression was not the goal of the CountTimeSeries package, but can be used for both a Poisson and a Negative Binomial regression. In R this is provided, for example, by the pscl package (Jackman [8]) with extensions by Zeileis et al. [9]. tscount can only be used for (Quasi-)Poisson regression, as it was designed only for Poisson and Quasi-Poisson INGARCH models.

In Julia, there is no package dealing with INGARCH and INARMA models, but like in R, there are packages for Hidden Markov models, like HMMBase, see Mouchet [10].

In the remainder of this paper, the CountTimeSeries package is first motivated and all its functionalities are explained. Then, four applications showcase how the package is used it practice. The first application deals with the spread of COVID-19 and focuses on the INGARCH framework and prediction. The second application analyzes the number of animals which were submitted to a New Zealand health laboratory and suffered from anorexia or skin lesions. Thinning-based INARMA models are used to shed more light on a discussion about the need of incorporating conditional overdispersion and zero inflation for this data set. During the analysis, inference is conducted on parameter estimates and information criteria used. Application 3 uses data first analyzed by Weiß and Feld [11] containing the number of corporate insolvencies in Rhineland-Palatinate. Thereby, the focus lies on the model choice, 54 models with different time trends and model specifications are fitted and information criteria used to select the best model. It is discussed whether there are spatial clustering effects in the characteristics of selected models and if there is a downwards trend in the number of insolvencies. Application 4 waives real data, a simulation study is carried out to assess the finite sample properties of Maximum Likelihood estimation for a Poisson INGARCH(1, 1). Different methods to initialize the conditional mean recursion, different parameter values, and different lengths of time series are compared. The simulation study is conducted in both Julia and R to compare the estimation time. Finally, Section 7 summarizes results, and gives an outlook and concludes.

## 2. The CountTimeSeries Package

When a new software package is developed, its rationale for existence should be addressed first. In other programming languages, there are many established packages to model count data time series. Three reasons spoke for the development of a new Julia package. Despite Julia’s strong growth in popularity during recent years, no package for count data existed. Methods covered by other programming languages should be provided for Julia users. The goal was not only to translate functions, but also to create a package that can be easily extended for other models and alternative estimation methods. Julia’s multiple dispatch feature allows adding model types and methods to existing functions for a unified notation. The third reason for the new package is its aforementioned advantages im terms of computing time. In the following, the general structure of the package is described in detail.

### 2.1. INGARCH Framework

By now, the package covers the INGARCH and INARMA framework and some important generalizations. The former was first described by Ferland et al. [2] as an integer process ${\left\{Y_{t}\right\}}_{t\in Z}$ with   

(1) $$\begin{array}{cc} Y_{t}|F_{t-1} & ∼\mathrm{Pois}(\lambda_{t})\\ \lambda_{t} & =\beta_{0}+\sum_{i=1}^{p}\alpha_{i}Y_{t-i}+\sum_{i=1}^{q}\beta_{i}\lambda_{t-i} \end{array}$$

where $F_{t}$ denotes the information set available at time *t*. Alternatively, the conditional distribution can be replaced by a Negative Binomial distribution with overdispersion parameter ϕ>0 such that the conditional variance becomes $\mathrm{Var}\left(Y_{t}|F_{t-1}\right)=\lambda_{t}+\lambda_{t}^{2}/\phi$.

Different extensions have proven useful in practice. Currently covered by the CountTimeSeries package are additional regressors, a log-linear link function, and zero inflation. In line with Liboschik et al. [4], regressors can be included in two different ways—as internal or as external regressors. By adding internal regressors $X_{t}^{\left(I\right)}$ in (1), the conditional mean becomes

(2) $$\begin{array}{c} \lambda_{t}=\beta_{0}+\sum_{i=1}^{p}\alpha_{i}Y_{t-i}+\sum_{i=1}^{q}\beta_{i}\lambda_{t-i}+\eta^{\prime }X_{t}^{\left(I\right)} \end{array}$$

To further include external regressors $X_{t}^{\left(E\right)}$, the conditional mean is defined as

(3) $$\begin{array}{cc} \lambda_{t} & =\nu_{t}+\eta^{\prime }X_{t}^{\left(E\right)}\\ \nu_{t} & =\beta_{0}+\sum_{i=1}^{p}\alpha_{i}Y_{t-i}+\sum_{i=1}^{q}\beta_{i}\nu_{t-i}+\zeta^{\prime }X_{t}^{\left(I\right)} \end{array}$$

In contrast to external regressors, internal regressors enter the recursive definition of conditional means. Often, it is not crucial how a regressor is declared by the user. The approach has been developed by Liboschik et al. [12] to model interventions that affect the observable process $Y_{t}$ without affecting the underlying dynamics of the mean recursion, see Liboschik et al. [12].

If one wants to replace the linear link function in above models with a log-linear link, Equation (3) become

(4) $$\begin{array}{cc} \log(\lambda_{t}) & =\nu_{t}+\eta^{\prime }X_{t}^{\left(E\right)}\\ \nu_{t} & =\beta_{0}+\sum_{i=1}^{p}\alpha_{i}\log\left(Y_{t-i}+1\right)+\sum_{i=1}^{q}\beta_{i}\nu_{t-i}+\zeta^{\prime }X_{t}^{\left(I\right)} \end{array}$$

If zero inflation should be included, only the first line in (1) is changed. Then, given $F_{t-1}$, $Y_{t}$ is assumed to follow a singular distribution at zero with probability ω and to follow a Poisson (or Negative Binomial) distribution with probability 1−ω. The conditional probability to observe a zero then is $P\left(Y_{t}=0|F_{t-1}\right)=\omega +\left(1-\omega \right)\exp\left\{-\lambda_{t}\right\}$. The CountTimeSeries package supports all combinations of these generalizations of the INGARCH model.

### 2.2. INARMA Framework

Besides the INGARCH framework, the CountTimeSeries package also covers another important model class widely used in count data analysis, INARMA models. Alzaid and Al-Osh [1] introduced an integer counterpart of AR(*p*) processes based on binomial thinning, which was then further developed and extended. The simple INAR(1) model is defined as $Y_{t}=R_{t}+\alpha \circ Y_{t-1}$, where $R_{t}$ is a latent, non-negative, and integer-valued process often called innovation or immigration process. The thinning operator “∘” is defined for a constant α∈[0,1] and a non-negative, integer random variable *X* as $\alpha \circ X=\sum_{i=1}^{X}Z_{i}$ for X>0, where $Z_{i}\overset{\mathrm{iid}}{∼}\mathrm{Bern}\left(\alpha \right)$ and zero if X=0.

In line with the notation of Weiß et al. [5], a non-negative and integer-valued process $\left\{Y_{t}\right\}$ follows INARMA(*p*, *q*) process if   

(5) $$\begin{array}{cc} Y_{t} & =R_{t}+\sum_{i=1}^{q}\beta_{i}\circ R_{t-i}+\sum_{i=1}^{p}\alpha_{i}\circ Y_{t-i}\\ R_{t} & ∼\mathrm{Pois}(\beta_{0}) \end{array}$$

The distribution of $R_{t}$ can also be chosen as a Negative Binomial distribution with mean $\beta_{0}$ and an overdispersion parameter ϕ, such that its variance is $\beta_{0}+\beta_{0}^{2}/\phi$. One way to include regressors in above model would be to add another additive component $Z_{t}$ to the first line, whose mean depends on regressors $X_{t}^{\left(E\right)}$. In the CountTimeSeries package, the distribution of $Z_{t}$ can be chosen as Poisson or as Negative Binomial. The regressors included that way are referred to as external regressors, as they do not affect the innovation process $R_{t}$. Another possible inclusion of regressors is implemented as internal regressors $X_{t}^{\left(I\right)}$. The Poisson INARMA(*p*, *q*) with regressors then becomes

(6) $$\begin{array}{cc} Y_{t} & =R_{t}+\sum_{i=1}^{q}\beta_{i}\circ R_{t-i}+\sum_{i=1}^{p}\alpha_{i}\circ Y_{t-i}+Z_{t}\\ R_{t} & ∼\mathrm{Pois}\left(\beta_{0}+\eta^{\prime }X_{t}^{\left(I\right)}\right)\\ Z_{t} & ∼\mathrm{Pois}\left(\zeta^{\prime }X_{t}^{\left(E\right)}\right) \end{array}$$

Note that the mean of $Z_{t}$ only depends on the regressors and no intercept parameter for identification. To include zero inflation, the distribution of $R_{t}$ is altered to be zero with probability ω and to follow a Poisson with probability 1−ω, see, for example, Aghababaei Jazi et al. [13]. This approach inflates the probability of $Y_{t}$ to be zero indirectly, in contrast to the approach for the INGARCH framework. The reason for it is found when taking a closer look at how the likelihood is computed for an INARMA model. As $Y_{t}$ is composed of multiple, non-observable parts, the distribution of $Y_{t}$ given past observations is the result of a convolution. A convolution simplifies from a computational point of view if possible values for each component are bounded. For the autoregressive part, each result of a thinning operation is bounded from above by the corresponding past observation. Including zero inflation as described above preserves the property $R_{t}\leq Y_{t}$, which would not hold if zero inflation was included in the same way as for INGARCH models.

### 2.3. Package Structure

The general structure of the package can be divided into four parts: model specification, generating data from a model, fitting a model to data, and prediction.

For the specification of a model, object types have been implemented. Starting from the top, the type CountModel covers every possible model described in the previous section. Models in the INGARCH or INARMA framework are collected in the types INGARCH and INARMA, respectively. Subtypes of these two are finally INGARCHModel, INARCHModel and IIDModel, as well as INARMAModel, INARModel, and INMAModel. This definition of a type tree allows to implement methods for certain groups of models.

A wrapper function Model() is implemented to specify a model. The user provides the model framework, INGARCH or INARMA, the distribution, the link function, model orders *p* and *q*, regressors if wanted with an indicator whether they should be treated as internal or external and whether or not zero inflation should be considered. Default setting is a simple Poisson IID model. A Negative Binomial INARCH(1) with zero inflation is for example specified by

Model(distr = "NegativeBinomial", pastObs = 1, zi = true).

To generate artificial data from a model, the specification is paired with parameter values. This can generally be done in two ways: as a vector of values or as a newly defined data type parameter, with entries for $\beta_{0}$, α, β, η, ϕ, and ω. Note that in the implementation, no notational distinction is made between parameters for internal and external regressors. The two ways of providing parameter values are useful for example during the optimization of the likelihood, which uses the parameter vector, whereas estimation results are more convenient to handle as the parameter type.

Once a model and parameter values are specified, data are generated with the simulate() function by calling simulate(T, model, parameter) for a time series of length T.

Before fitting a model to a time series, settings for the maximization of the likelihood can be provided. The user can specify initial values and the method for the optimization routine with the MLESettings() function. The optimization routine can be "NelderMead", "BFGS", or "LBFGS". If inference in terms of confidence intervals shall be conducted, the argument ci needs to be set to true. Standard errors are then computed from the numerical Hessian matrix.

Estimation of parameters is carried out by the fit() function, which takes the time series, the model, and, if chosen, the settings as input. The likelihood is maximized while considering constraints on the parameters. Constraints include positivity of conditional means at any time, proper thinning probabilities and constraints that ensure stability of the process.

The function parametercheck checks whether parameters are valid whenever calling the log-likelihood function. If invalid parameters are put in, the log-likelihood function simply returns negative infinity. This approach is usually unproblematic if starting values for the optimization are valid and not too close to being invalid.

The fit() function returns an object with estimates, standard errors, log-likelihood for estimates, and many more. This result object can be forwarded to functions for information criteria, AIC(), BIC(), and HQIC(), or to the function pit(). Then, the non-randomized probability integral transform histogram (see Czado et al. [14]) is plotted.

A forecast can be carried out by the predict() function. For models of the INGARCH framework, two options are available. Predictions can either be deterministic or simulation-based. In the deterministic approach, conditional means are used as prediction, and if an observation in the definition of the conditional mean is not observed, it is replaced by its corresponding prediction. In the simulation-based approach, the time series is continued many times with random realizations, or chains, following the process. This conveniently provides prediction intervals as the quantiles of the chains.

Assembling all parts together, this section is closed with an example of a Poisson INARCH(1). The code to specify the model, simulate a time series of length 500 with parameters $\beta_{0}=3$ and $\alpha_{1}=0.95$, fitting and predicting 100 steps into the future with prediction intervals from 10,000 chains would be

model = Model(pastObs = 1)

y = simulate(500, model, [3, 0.95])[1]

result = fit(y, model)

pred = predict(result, 100, 10000)

## 3. Application: COVID-19

For the first application, we take a closer look at the spread of COVID-19 in Germany. The global pandemic has moved disease spread into public focus. Being able to predict the number of newly infected patients is a cornerstone for policy making and resource planning in hospitals and medication supply. Many sophisticated models have been developed to predict the progression of infection rates. In the following, the CountTimeSeries package is utilized to conduct such predictions with a rather simple INGARCH model and daily data from German administrative districts. As an example, the application focusses on the number of new infections in Limburg-Weilburg, which is the district with a population density closest to the nationwide population density.

Data on infections of contagious, notifiable diseases are collected from the districts health authorities by the Robert–Koch Institut, see RKI [15]. The data set used in the course of the following analysis was prepared and published by NPGEO [16]. Besides general information on districts it contains the daily number of newly reported COVID-19 cases for all 401 German administrative districts on the NUTS 3 resolution. The observed time ranges from 7 January 2020 to 15 March 2021, totaling 434 days.

A first look at the data for the district of Limburg-Weilburg reveals that ~35% of observations are zero and 9% of observations exceed 50, which underlines the suitability and importance of a count data model. The excess of zeros in the data is partly explained by the beginning of the pandemic with only few cases and the rather low counts in general are due to the fine resolution in both, the time and space domain.

While advanced approaches for disease modeling incorporate many driving factors, this analysis relies purely on the number of infections in Limburg-Weilburg and its neighboring districts. Demographic factors, holidays and school closings, contact limitations, introduction of mandatory face masks, and occupancy of hospitals and care facilities are left out.

### 3.1. Model

**Figure 1:**
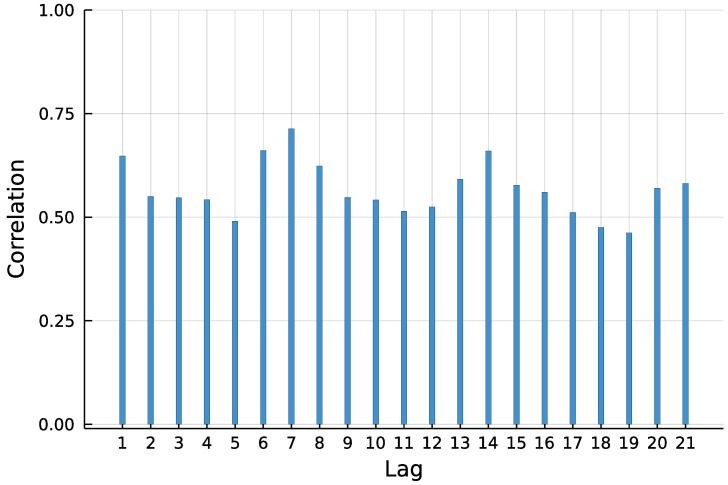
Autocorrelation function (ACF) of daily new infections in Limburg-Weilburg.

To predict the number of new COVID-19 infections in Limburg-Weilburg, the widely used INGARCH(1, 1) model with log-linear link is chosen as a starting point. According to the World Health Organization [17], the incubation time of COVID-19 is on average five to six days. It is further stated that virus transmission occurred more often from patients with symptoms in empirical studies. Therefore, and to capture the weekly seasonality pattern visible in Figure 1, the lag order 7 is included in the model as well.

In the time series, the mean count is around 14 whereas the variance is 642. This marginal overdispersion does not necessarily indicate that a Negative Binomial must be considered as conditional distribution. The Poisson INGARCH processes with large dependency, meaning a sum of $\alpha_{i}$ and $\beta_{i}$ parameters close to one, can exhibit such an overdispersion. In the following, three different approaches are considered and compared. Besides a Poisson and a Negative Binomial model, a retrospective estimation of the overdispersion parameter after fitting a Poisson model, as, for example, described by Christou and Fokianos [18], is also considered. Let the sequence ${\hat{\lambda }}_{t}$ be the conditional means on the basis of estimates from a Poisson fit, *T* the number of observations, and *m* the number of parameters. The retrospective estimate of the overdispersion parameter ϕ is then given by solving the moment equation   

(7) $$\begin{array}{c} \sum_{t=1}^{T}\frac{{\left(Y_{t}-{\hat{\lambda }}_{t}\right)}^{2}}{{\hat{\lambda }}_{t}(1+{\hat{\lambda }}_{t}/\phi )}=T-m \end{array}$$

for ϕ. The data-generating process is assumed to be a Negative Binomial process, whereas all parameters apart from ϕ are estimated by fitting a Poisson model. Therefore, this approach falls into the class of Quasi-Maximum Likelihood estimation with additional moment based estimation of ϕ. In the following, this approach is referred to as a Quasi-Poisson approach.

**Figure 2:**
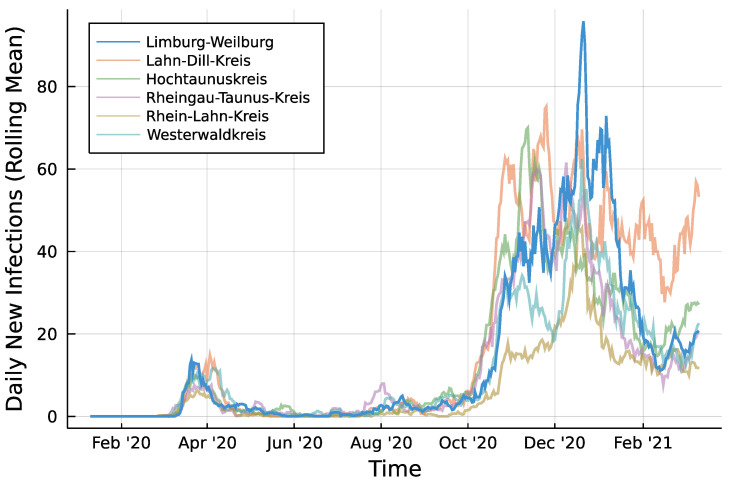
Daily infections in Limburg-Weilburg and Neighbouring Districts—7 day rolling mean.

Infection counts of neighboring districts from seven days prior are included to account for potential spillover effects between districts. For each of Limburg-Weilburgs five neighbouring districts—Lahn-Dill-Kreis, Hochtaunuskreis, Rheingau-Taunus-Kries, Rhein-Lahn-Kreis, and Westerwaldkreis—and one internal regressor is added. Figure 2 displays the rolling means of infection counts for all districts under consideration with a window size of one week. Let $N_{i,t}$ denote the number of new infections in neighboring district *i* at time *t*. In the same fashion as for the autoregressive part, regressors are included as $\log(N_{i,t}+1)$ and collected in $X_{t}$. Then, the model to be fitted can be summarized according to Equation (8) for the Poisson case.

(8) $$\begin{array}{cc} Y_{t} & ∼\mathrm{Pois}(\lambda_{t})\\ \log(\lambda_{t}) & =\beta_{0}+\alpha_{1}\log\left(Y_{t-1}+1\right)+\alpha_{7}\log\left(Y_{t-7}+1\right)+\beta_{1}\log\left(\lambda_{t-1}\right)+\zeta^{\prime }X_{t} \end{array}$$

### 3.2. Implementation

First step in the Julia implementation from above is to specify the models. With the regressors collected in matrix X, the Poisson model is created by running

modelPois = Model(model = "INGARCH",

pastObs = [1, 7],

pastMean = 1,

distr = "Poisson",

link = "Log",

external = fill(false, 5),

X = X)

For the alternative model, the distribution is simply replaced by "NegativeBinomial". To fit the models to the time series y, settings are chosen as the Nelder–Mead optimization routine and no inference. Then, the Poisson model is fitted by running

settingPois = MLESetting(y, modelPois, inits, optimizer = "NelderMead",

ci = false)

resultsPois = fit(y, modelPois, settingPois)

and for the Negative Binomial model accordingly. The retrospective estimation of the overdispersion parameter for the Quasi-Poisson approach is done by QPois(resultsPois). The function returns an updated results object.

In the last step, simulation-based prediction is performed using the function predict. For each of the three results, a matrix of new values for regressors is needed and saved as xNew. Then, the Julia code for a prediction with 10,000 chains is

predict(resultsPois, 7, 10000, xNew)

The application starts with using the first 322 observations until 30 November 2020 for estimation of parameters and predicting the following week. Successively, one further observation is used for estimation and another prediction performed until the first 420 observations are used for estimation.

### 3.3. Results

**Figure A1:**
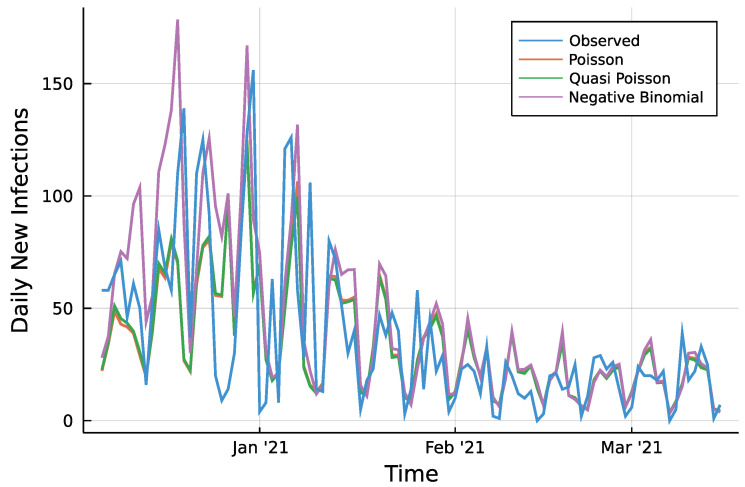
7 days ahead prediction and observed counts.

To assess the predictions, a first look reveals only minor visual differences between Poisson fit and the Quasi-Poisson approach as seen in Figure A1 in the Appendix A. Table 1 summarizes two different accuracy measures and the percentage of observed values which lie inside the prediction intervals. For every prediction horizon h=1,2,…,7, the root mean squared prediction error (RMSPE) is given. Further, the median absolute prediction error (MedAPE) is given to account for few large deviations that may influence the RMSPE. For both measures and all three approaches, one can see that a larger prediction horizon does not affect the accuracy in a negative way. The predictions even tend to be closer to the observed values for a larger horizon. The Quasi-Poisson approach is slightly more accurate compared to the Poisson approach. The absolute difference of prediction and observed counts is smaller than 10 in ~50% of cases for the two approaches regardless of the horizon. The Negative Binomial model appears to be less accurate compared to the other two. One reason for it might be the additional parameter during the maximization of the likelihood. This might cause a higher uncertainty in the estimation of all parameters and thus a less precise prediction. The difference between a Poisson model and the Quasi-Poisson can be seen by the percentage of observations inside the corresponding 95% prediction intervals. The Poisson case is far away from the desired 95%, prediction intervals are too narrow. The model simply can not account for the conditional overdispersion present in the data. The Quasi-Poisson and the Negative Binomial model are able to account for it.

To put these accuracy measures into context, the mean number of new infections being predicted is around 40. None of the three approaches can be considered very precise. However, this application showed that the Quasi-Poisson estimation can combine the advantages of a Poisson model and the Negative Binomial model.

In addition to the prediction results, Table 2 displays estimates, standard errors, and 95% confidence intervals when considering the complete data set in the estimation. On the left hand side, standard errors are computed from the Poisson model. The estimate of ϕ stems from the Quasi-Poisson approach. One can see that both autoregressive parameters $\alpha_{1}$ and $\alpha_{7}$ are significant, whereas the parameter $\beta_{1}$ is not. An INARCH model might be an alternative to the model considered here. The spillover effects are negative only for the first neighboring district, but not significant. The remaining four are significant and positive. The same can be found in the Negative Binomial case. In contrast to the Poisson model, neither the parameter $\beta_{1}$ nor both autoregressive parameters $\alpha_{1}$ and $\alpha_{7}$ are significant.

These findings hint at the existence and importance of including spillover effects in an epidemiological model. However, they do not prove any causal relationship, as the the models lack important driving factors of disease transmission. For more reliable results, a multivariate model or spatiotemporal model with all districts jointly enables to include spillover effects in both directions and is better suited for such epidemiological applications.

## 4. Application: Animal Health in New Zealand

Aghababaei Jazi et al. [13] were the first to analyze submissions to animal health laboratories in New Zealand. The data contain the monthly number of submissions with anorexia and skin lesions from 2003 to 2009. The authors use a zero inflated Poisson distribution for the innovations, where the probability of $R_{t}=0$ is inflated by a parameter ω. For the time series of animal submissions with skin lesions, this zero inflated INAR(1) process yields a significantly better fit compared to a simple INAR(1).

Mohammadpour et al. [19] argue that the empirical overdispersion in the data cannot be captured by the Poisson distribution. A test developed by Schweer and Weiß [20] was applied and reveals that this overdispersion is significant at the 5% level. To incorporate it, the authors suggest to use either a Negative Binomial distribution or the approach developed by them.

### 4.1. Model and Implementation

**Figure 3:**
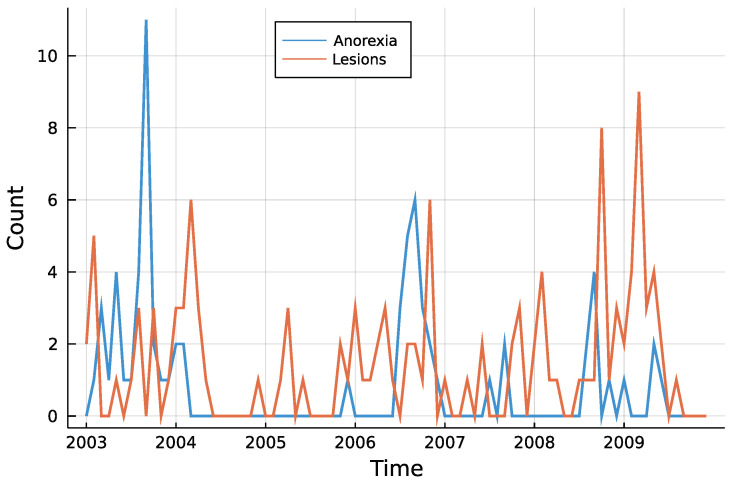
Monthly submissions with anorexia or skin lesions.

**Figure 4:**
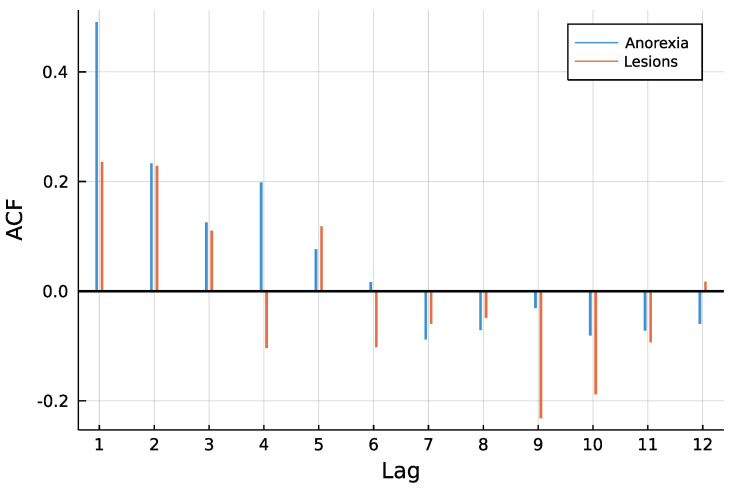
ACF of time series.

Figure 3 shows both time series. One can see that counts are rather low, a trend is not visible and especially for anorexia data, many observations are zero. The autocorrelation function of both time series in Figure 4 does not speak against a low-order INAR model. Eight different models are compared for the data, an INAR(1) and an INAR(2) model with both a Poisson and a Negative Binomial distribution, and for all of these four once with and once without zero inflation. Other regressors are left out, making the choice of a link function dispensable.

The first step in the analysis is to define the eight models, for example, a Poisson INAR(1) as

Model(model = "INARMA",

pastObs = 1,

zi = true)

making use of default arguments in the function. If a Negative Binomial shall be chosen, one simply needs to add distr = "NegativeBinomial". For a model order p=2, pastObs = 1 is changed to pastObs = 1:2.

With starting values for optimization saved as a vector inits of type parameter, the function MLESettings is again used to specify estimation settings as

settings = MLESettings.(fill(dat.Anorexia, 8), models,

optimizer = "NelderMead", ci = true)

The dot behind a function name indicates that it shall be applied element-wise, in our case on the vector inits. The output then is a vector of estimation settings. Following that manner, the results for both time series can be computed all at once by

results = Array{INARMAResults, 2}(undef, (8, 2))

results[:, 1] = fit.(fill(dat.Anorexia, 8), models, settings)

results[:, 2] = fit.(fill(dat.Lesions, 8), models, settings)

If one is now interested in diagnostics, for example, an information criterion, functions can be applied on this array of results as well. In case of the AIC, the user runs

AIC.(results, 2)

At this point, one needs to keep in mind that information criteria are based on the likelihood. When computing the likelihood, the number of observations to condition on varies with the autoregression order. Especially for rather short time series, this property needs to be accounted for. In the above computation of the AIC, the second argument indicates that for each model, the contribution of the first two observations to the likelihood are ignored making information criteria comparable.

### 4.2. Results

Table 3 summarizes the results of the estimation including zero inflation. Besides estimates, standard errors from the Hessian and the AIC are shown. For parameters $\beta_{0}$, $\alpha_{1}$, $\alpha_{2}$, and ω, colors indicate the significance, where dark green refers to significance at the 0.1% level, medium green at 1%, and light green at 5%. The *p*-value of the overdispersion parameter ϕ is ignored, as it can not be zero. In addition, Table A1 in the Appendix A shows results for all models without zero inflation.

Mohammadpour et al. [19] analyze the data on lesions and find that a Poisson distribution does not capture the overdispersion. The results below confirm these findings in terms of the AIC. For an INAR(1) and an INAR(2), the Negative Binomial dominates the Poisson distribution. Moreover, including the lag order p=2 lowers information criteria for both distributions and the estimates of $\alpha_{1}$ are not significant at the 5% level in that case.

Aghababaei Jazi et al. [13] emphasize the benefit of incorporating zero inflation in the innovation process for the analysis of animal submissions with skin lesions. Only when considering the Poisson models, this is supported by the highly significant zero inflation parameter estimates and a lower AIC. However, when a Negative Binomial is used, the zero inflation parameters are not significant. In fact, dropping zero inflation for the NB-INAR(2) yields an even lower AIC. The results are similar for Anorexia data, although estimates of $\alpha_{2}$ are not significant at 5%, both INAR(2) models have a lower AIC than the corresponding INAR(1).

In conclusion, this study has confirmed the findings of Mohammadpour et al. [19]. When only looking at first-order INAR models, there is a conditional overdispersion in the number of animal submissions with skin lesions that cannot be captured by a Poisson distribution. The findings of Aghababaei Jazi et al. [13] can also been confirmed. In case of a conditional Poisson distribution, it is useful to include zero inflation. The need for a zero inflation vanishes in the example when switching to the Negative Binomial. This is comprehensible, as the probability of a Negative Binomial being zero is larger than the probability of a Poisson with the same mean being zero. Therefore, switching to a Negative Binomial also inflates the zero probability. Although this application is only one specific example, we can draw general conclusions from it: The need to extend a given model during an application highly depends on the model you start with. When choosing a model, it can be useful to cover a broader range of potential models to uncover patterns. Especially for higher-order INARMA type models, the computer intensive evaluation of the likelihood makes such broad comparisons cumbersome. However, the CountTimeSeries package provides an efficient implementation of the likelihood and makes such broad comparisons feasible.

## 5. Application: Corporate Insolvencies in Rhineland-Palatinate

After both model frameworks are introduced, the focus is now put on model choice and diagnostics. Weiß and Feld [11] were the first to analyze corporate insolvencies in Rhineland-Palatinate, one of the 16 federal states in Germany. The data were made available as supplementary material and are used in the scope of the following. Rhineland-Palatinate consists of 36 administrative districts at the NUTS-3 level: 24 rural districts and 12 district-free cities. For each of these districts, the monthly number of corporate insolvencies between 2008 and 2016 is observed. The data are used to investigate whether there is a downwards trend in insolvency numbers. Röhl and Vogt [21] state that this trend is especially visible after the financial crisis.

### 5.1. Models and Implementation

**Figure 5:**
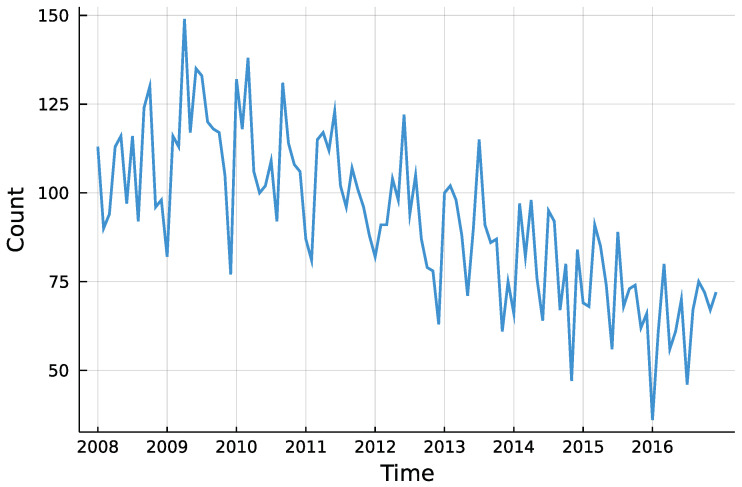
Total number of monthly insolvencies in Rhineland-Palatinate.

The question whether there was a trend in insolvencies is going to be answered for each district individually. This enables to descriptively check for regional differences in insolvency counts. Although insolvencies are presumably influenced by macroeconomic factors, the only external influence incorporated here is the potential trend. Besides that, the process is assumed to be self-driven. Looking at the total counts in Figure 5, one can see the downwards trend starting around 2010, as Röhl and Vogt [21] state. Further, there seems to be no seasonality pattern in the time series.

Weiß and Feld [11] investigated the model choice considering IID data with and without a linear trend and either a Poisson or Negative Binomial distribution. Here, we include IID data and both frameworks, INGARCH and INARMA. Potential orders are chosen to be (1, 0), (2, 0), (1, 1), and (2, 1) to capture serial correlation if present. Both distributions, Poisson and Negative Binomial, are incorporated. A trend component may be omitted, included for the complete range of time, or starting in January 2010. Thereby, all link functions are chosen to be a log-linear link. That way, negative coefficients of the trend component are no threat to the restriction of positive conditional means. This sums up to 54 potential models to be estimated for each of the 36 time series. To save computation time, inference is only conducted for chosen models and not for all 1944 combinations of models and time series. In the package, standard errors are computed via the numerical Hessian.

After the estimation, information criteria can be computed by running AIC(results), BIC(results), or HQIC(results). An additional argument dropfirst can be passed to these functions to suppress the likelihood contributions of first observations. This comes handy when comparing for example an INARCH(1) and an INARCH(2). Likelihood based estimation of these conditions on the first one or two observations respectively. For rather short time series, this difference might be crucial.

To check if a choice of model is suitable, the non-randomized probability integral transform (PIT) histogram is used. It was developed by Czado et al. [14] for count data time series and does not rely on random numbers. The packages function pit produces a histogram running pit(results, nbins = 10, level = 0.95) that is uniformly distributed if the model choice is correct. The argument nbins specifies the number of bins and the argument level can be used to test the uniform distribution at the (1−level)-level. If level is put in, lines are drawn in the histogram and if at least one bin exceeds the lines, the null hypothesis of a uniform distribution is rejected.

In the analysis, potential models are defined and fitted to all 36 time series. Then, the best model is chosen for each district and verified by the packages diagnostic tool.

### 5.2. Results

In the fitting of 54 models to each of the 36 districts, all optimization procedures converged. For districts with a marginal underdispersion, all models with a Negative Binomial distribution have not been fitted. Inference has not been conducted so that results purely rely on the model selection criterion. Table A2 summarizes the model selection for all 36 districts together with mean and variance of time series. Many of the models selected exhibit no serial dependence. In these cases, and as is to be expected, the Negative Binomial was selected when the variance is clearly larger than the mean.

**Figure 6:**
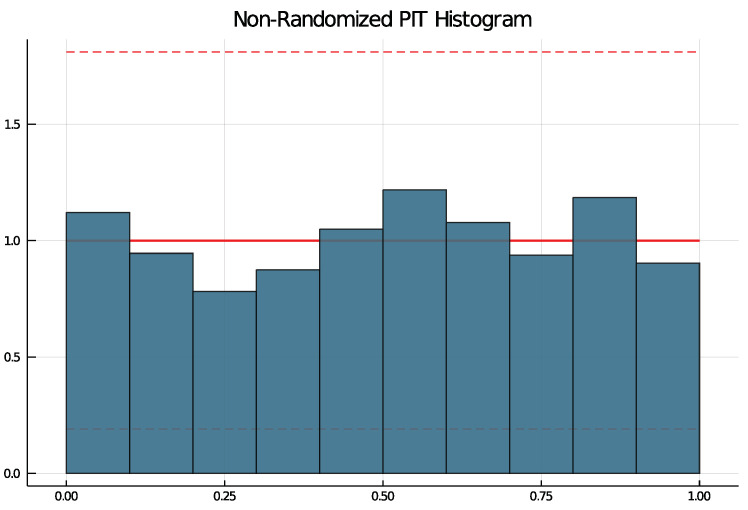
PIT histogram for Trier.

All selected models have been checked with the non-randomized PIT histogram. None of the selected models produce a PIT histogram for which the null hypothesis of a uniform distribution was rejected. Figure 6 shows an example PIT histogram for Trier, where the dashed lines represent critical values for the height on bins for which, if exceeded, the null hypothesis would be rejected. The confidence bank is rather wide due to few observations.

**Figure A2:**
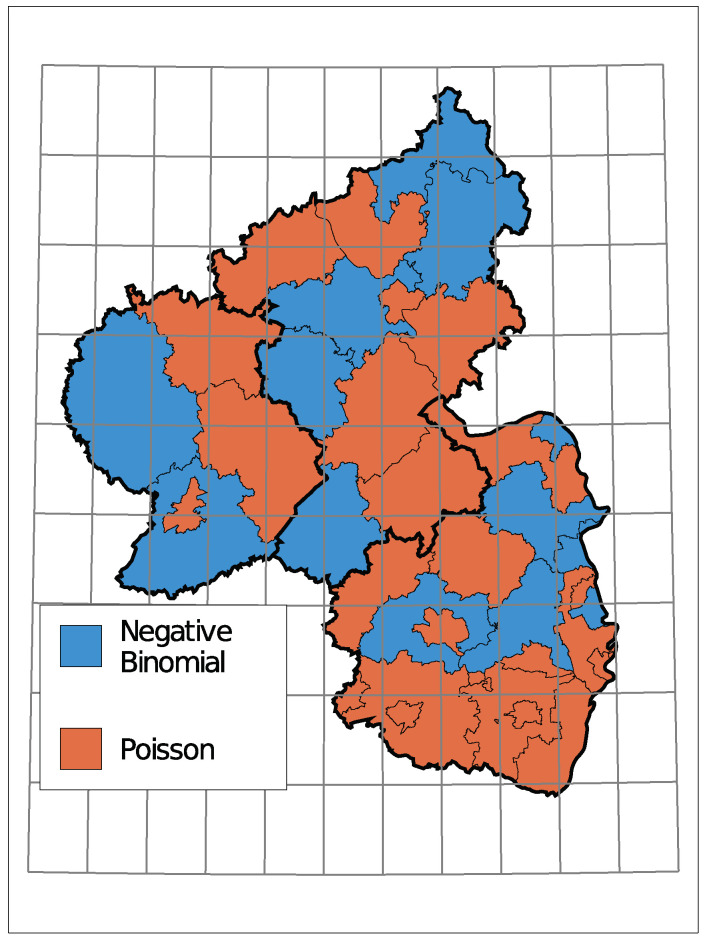
Selected distribution.

The (conditional) distribution is an indicator of how much the insolvency count varies compared to its mean. Figure A2 in the Appendix A displays the selected distributions where a clustering of districts with the same distribution is only vaguely recognizable.

**Figure 7:**
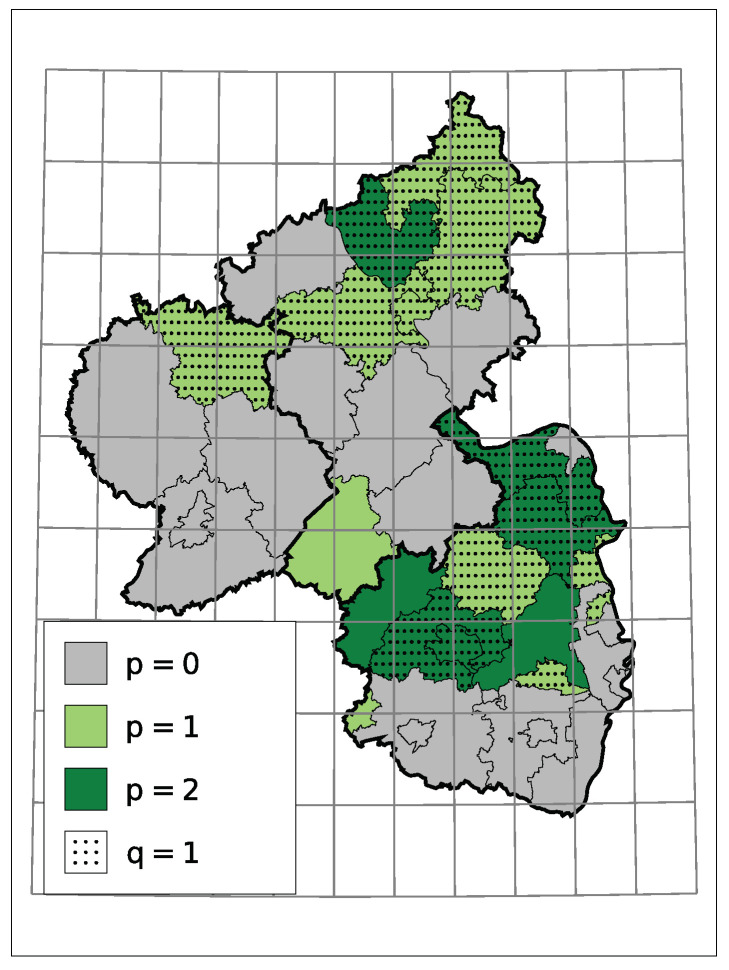
Selected Model Order.

A non-zero model order *p* or *q* implies a serial dependence besides a potential trend. In the case at hand, for districts with serial dependence, all estimates indicate that a larger/smaller number of insolvencies in one month tends to go along with a larger/smaller number in the month after. One reason for it can be an interconnected economy within the district, but not necessarily. Macroeconomic factors can also cause such dependence. Figure 7 displays the selected models orders. One can see that those districts with dependence seem to cluster.

**Figure 8:**
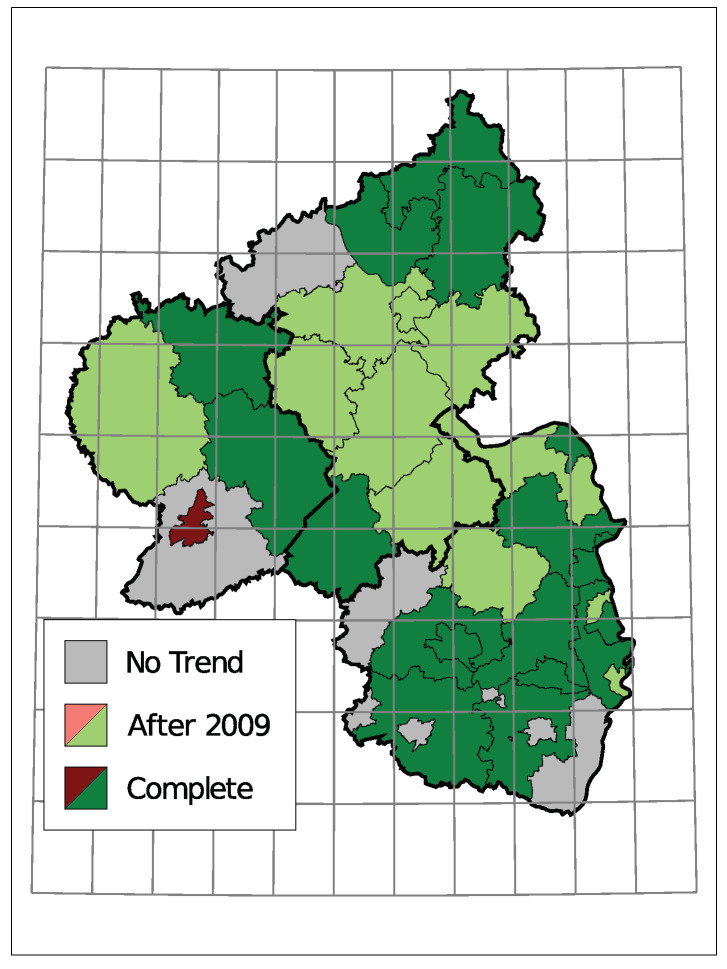
Selected Trend: upwards (red) or downwards (green).

The selected time trends and the sign of the corresponding coefficient are summarized in Figure 8. A grey district has no trend component in the selected model, light colors represent districts with a trend starting in 2010 being selected, and dark colors represent a selected trend along the complete observed time. Thereby, districts are colored green for a downwards trend in the insolvency counts and red for an upwards trend.

Only one district, the urban area of Trier, exhibits an upwards trend. Seven districts do not have a trend in the selected model and the remaining 28 show a downward trend. Districts in the center of Rhineland-Palatinate and the district far east tend to have a trend component starting in 2010. A complete trend was chosen more often in the southern region and the north.

This analysis did not incorporate any macroeconomic variables to explain the insolvencies and also no information about the companies being bankrupt, yet it showed that characteristics in terms of dispersion, autocorrelation and time trend cluster spatially. The results suggest that there could be an interconnection between districts and that it would be interesting to conduct the study in a multivariate setting instead of looking at districts individually.

## 6. Simulation Study: Finite Sample ML—Estimation

In many applications, the length of count data time series is rather short. Consistency and asymptotic normality are proven for Maximum Likelihood estimates of an INGARCH processes parameters, but a potential finite sample bias is often not addressed.

This last application pursues two goals. On the one hand, finite sample properties of a Maximum Likelihood estimation are investigated in a simulation study. On the other hand, the analysis is performed with both the CountTimeSeries package in Julia and the tscount package in R. Computation time for the estimation is compared between these two.

### 6.1. Study and Implementation

The general strategy for this study is to generate time series following an INGARCH process and then estimate its parameters by Maximum Likelihood. The study focusses on Poisson INGARCH(1, 1) processes with identity link, as it is commonly used in applications. The general framework covered by the CountTimeSeries package as described in Section 2 thus simplifies to

$$\begin{array}{cc} Y_{t}|F_{t-1} & ∼\mathrm{Pois}(\lambda_{t})\\ \lambda_{t} & =\beta_{0}+\alpha_{1}Y_{t-1}+\beta_{1}\lambda_{t-1} \end{array}$$

Every combination of parameters $\alpha_{1},\beta_{1}\in \left\{0.1,0.2,\ldots ,0.8\right\}$ with a sum smaller than 1 is incorporated in this study, which ensures stationarity. The intercept is chosen in such a way that the marginal mean $\beta_{0}/\left(1-\alpha_{1}-\beta_{1}\right)$ equals 15 for every choice of $\alpha_{1}$ and $\beta_{1}$. Then, for each set of parameters, a total of 1000 time series of lengths T∈{50,200,1000} are generated and its parameters estimated with four slightly different methods. The conditional mean recursion $\lambda_{t}$ above can be initialized with either the marginal mean, the first observation or the intercept in the CountTimeSeries package. The fourth possibility, not covered by both packages, is to treat the initial value of the recursion as additional parameter and estimate it.

Time series can be generated with the CountTimeSeries package by running

simulate(T, model, truepar)

for a model specification model and parameters truepar. Then, with estimation settings collected in the object setting, parameters are estimated by

fit(y, model, setting, initiate = initiate, printResults = false)

The optional argument initiate specifies how the recursion $\lambda_{t}$ is started and the argument printResults can be set to false to omit that results are printed in the console.

### 6.2. Results

**Figure 9:**
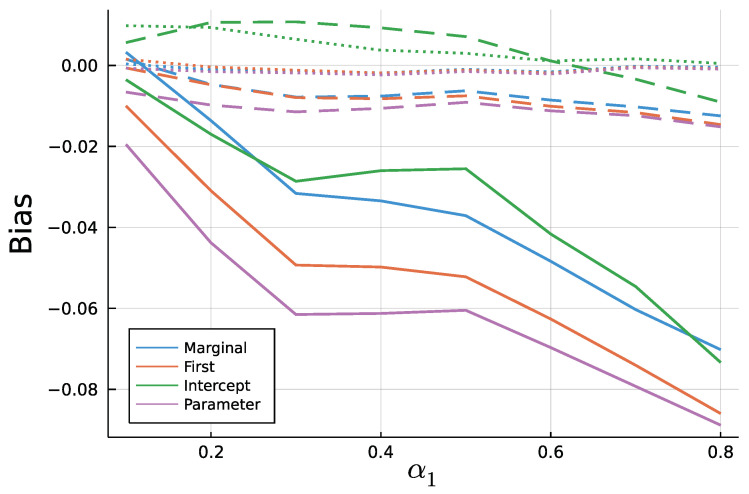
Mean Bias of ${\hat{\alpha }}_{1}$ for T = 50 (solid), T = 200 (dashed), and T = 1000 (dotted).

**Figure 10:**
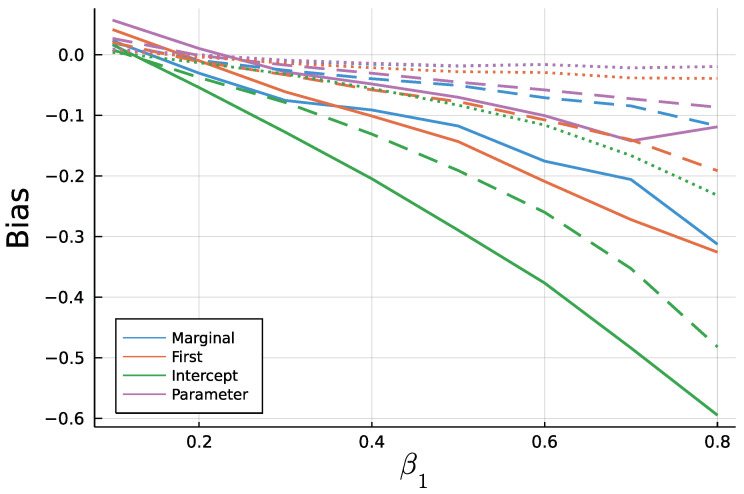
Mean Bias of ${\hat{\beta }}_{1}$ for for T = 50 (solid), T = 200 (dashed), and T=1000 (dotted).

Figure 9 and Figure 10 display the mean bias of estimates $\alpha_{1}$ and $\beta_{1}$, respectively, and negative values thereby indicate an estimate smaller than the true value. Note that for a true value of 0.1, there are eight different parameter combinations aggregated in the bias, but only one combination for a true value of 0.8 on the x axis due to the stationarity restriction. Initializing the recursion with the marginal mean and the first value appear to yield similar biases. This is a plausible result, considering that the first observation should equal the marginal mean on average. Treating the first conditional mean as parameter also gives similar biases in case of moderate (T=200) and long (T=1000) time series. In case of short time series T=50, this approach exhibits the largest absolute bias for $\alpha_{1}$ while at the same time being the least biased for $\beta_{1}$. Initializing the recursion with the intercept seems to perform poorly especially for the estimate of $\beta_{1}$ for larger true values of $\beta_{1}$. In general, the absolute bias should be larger for larger sums of $\alpha_{1}$ and $\beta_{1}$, as the deviation between intercept and expectation of $\lambda_{1}$ is larger.

**Figure A3:**
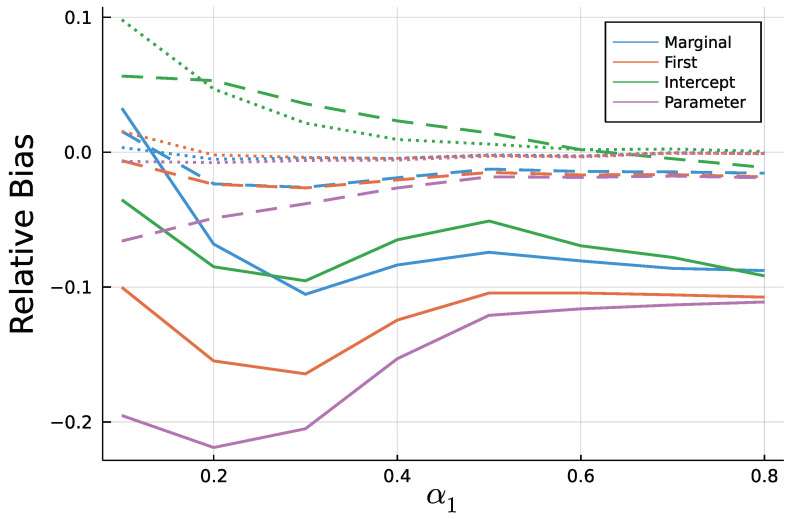
Mean relative bias of ${\hat{\alpha }}_{1}$ for T = 50 (solid), T = 200 (dashed), and T = 1000 (dotted).

**Figure A4:**
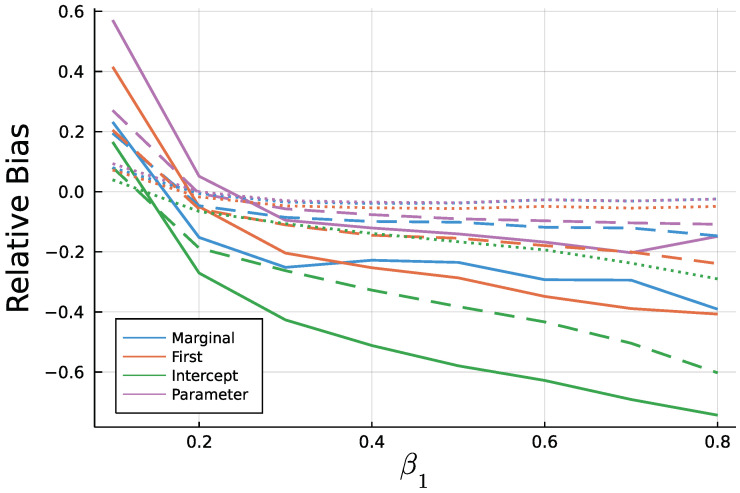
Mean relative bias of ${\hat{\beta }}_{1}$ for T = 50 (solid), T = 200 (dashed), and T = 1000 (dotted).

As to be expected by the consistency of Maximum Likelihood estimates, longer time series go along with smaller biases. In addition to Figure 9 and Figure 10, Figure A3 and Figure A4 in the Appendix A show relative biases. Thereby, biases are scaled by the true value of the parameter to be estimated. It reveals that the estimate $\alpha_{1}$ exhibits larger relative biases for smaller true values.

Only considering the first three initialization methods, one single estimation took around 0.003 s for T=50, 0.007 s for T=200 and 0.02 s for T=1000 on average in Julia. The initialization method does not affect the computation time while larger values of $\alpha_{1}+\beta_{1}$ increased the computation time. Using the R package tscount, estimation took on average 0.11, 0.18 and 0.55 s for the three lengths of time series and thus around 25 times longer.

Conclusively, this simulation study revealed the importance of a well chosen initialization method as well as systematic biases for Maximum Likelihood fitting of short INGARCH(1, 1) time series. Starting the mean recursion with the intercept should be avoided. Treating the first conditional mean as parameter might be the least restrictive among the four methods, but as an additional parameter, it might add noise to the estimation of the other parameters. It is recommended to initialize the mean recursion by the processes marginal mean or the first observed value in case of an INGARCH(1, 1), since the biases are comparable to the parameter initializer. However, for some processes in the INGARCH framework, for example an INGARCH(1, 1) with log-linear link, computation of the marginal mean is computer intensive or must be approximated. Therefore, starting the recursion with the first observation appears to be a good choice. It is also the default setting in the CountTimeSeries package.

## 7. Discussion and Outlook

Three real-life applications and the simulation study were chosen to give a broad overview on the CountTimeSeries package. All functions of the package have been shown: specifying a model, generating time series, estimating parameters, using information criteria, as well as a diagnostic tool and forecasting. In the spirit of the Julia programming language, the package is easy to learn, covers a broad range of models, and is fast compared with interpreted languages. It is the first software package that covers both widely used frameworks, INGARCH and INARMA processes.

Application 1 showed how the CountTimeSeries package can be used to investigate complex non-stationary time series from epidemiology and compute forecasts. Comparing prediction accuracy the prediction intervals, it revealed the advantage of the Quasi-Poisson approach. Application 2 and 3 showed how to fit multiple models to data simultaneously thanks to element-wise application of functions. In the second application, a contribution was made to an existing discussion about the need to incorporate zero inflation and overdispersion in a model for animals with skin lesions in New Zealand. Although the results of the previous literature have been confirmed, increasing the model order has put these findings in a different light.

Application 3 used corporate insolvency data to analyze if model choices based on information criteria cluster spatially. In line with previous literature, a downwards trend in the number of insolvencies was visible for most districts in Rhineland-Palatinate. Districts where this trend started in 2010 after the financial crisis appeared to come in clusters. Similar clustering effects were visible for dispersion characteristics and the order of dependency.

The simulation study in the end showed that there is a systematic bias when fitting an INGARCH(1, 1) model to a rather short time series. Thereby, it was revealed that one should not initialize the conditional mean recursion by the intercept. In comparison with R, the Julia package was faster, making broad simulation studies computationally more feasible.

Although the CountTimeSeries package already covers many important models, some extensions would be useful supplements. A function to compute the mean, variance, and ACF of a given model and parameters would first enable to compare empirical moments and theoretical moments from estimation results. In applications this might reveal weaknesses in the model choice. Further, such a function could be used to estimate parameters in a GMM approach. Especially for higher-order INARMA models, likelihood evaluation is computationally expensive. GMM might enable estimation in acceptable runtime. For models with no closed form moments, a simulation-based approximation of moments could be an alternative. If GMM estimation was integrated into the package, the computation of empirical moments could easily be replaced by robust moment estimators, like trimmed means, to provide robust estimation techniques.

When dealing with real-life data, extreme observations, not following the usual model, are commonly found. Other approaches to account for possible extreme observations include classical M-estimators or the recently published method by Li et al. [22] or approaches based on the density power divergence, see for example Xiong and Zhu [23].

Another family of models that would enrich the Julia package are models with bounded counts, see for example Weiß [24]. Examples for the usefulness of such models include time series or cross sectional data on product ratings or the number of rainy days in a month. An approach to include zero inflation for such models is discussed by Möller et al. [25]. As an alternative to zero inflation as presented here, would be hurdle models to account for a surplus of zeros.

Above extensions match the notation and the current infrastructure of the package and thus do not need a substantial reconstruction of the package. An inclusion of multivariate models and spatiotemporal models brings along new challenges for the inclusion in the Julia package, but would be rewarding for many applications. The COVID-19 application discussed above is only one example where a multivariate model is useful. Modeling multiple interacting count data processes jointly is also frequently used in finance, see, for example, Quoreshi [26].

## Figures and Tables

**Table 1:** Prediction Limburg–Weilburg: Root Mean Squared Prediction Error, Median Absolute Prediction Error and Percentage of Observations Inside the 95% Prediction Interval.

Criterion	Model	Prediction Horizon						
		1	2	3	4	5	6	7
RMSPE	Poisson	29.53	30.18	30.11	30.14	28.81	28.07	27.86
	Quasi-Poisson	29.53	30.07	30.04	29.98	28.59	27.89	27.71
	Negative Binomial	30.95	31.14	30.89	30.86	30.38	29.68	30.09
MedAPE	Poisson	10.14	10.39	10.19	10.27	10.38	10.20	10.03
	Quasi-Poisson	10.07	10.22	10.26	10.12	10.28	9.96	9.69
	Negative Binomial	12.55	12.43	12.19	12.05	11.71	11.97	11.75
Inside PI	Poisson	46.5	46.5	46.5	48.5	50.5	49.5	49.5
	Quasi-Poisson	97.0	97.0	97.0	97.0	97.0	97.0	97.0
	Negative Binomial	98.0	98.0	99.0	99.0	99.0	99.0	99.0

**Table 2:** Estimation Results, standard errors, and 95% confidence intervals.

	(Quasi-)Poisson			NegativeBinomial		
	**Estimate**	**Std. Err.**	**Conf. Interval**	**Estimate**	**Std. Err.**	**Conf. Interval**
$\beta_{0}$	−0.159	0.049	(−0.254,−0.063)	−0.540	0.107	(−0.749,−0.330)
$\alpha_{1}$	0.041	0.018	(0.006, 0.077)	0.115	0.063	(−0.010,0.239)
$\alpha_{7}$	0.122	0.022	(0.079, 0.165)	0.088	0.080	(−0.068, 0.245)
$\beta_{1}$	0.053	0.030	(−0.006, 0.112)	0.005	0.100	(−0.190, 0.200)
$\zeta_{1}$	−0.032	0.015	(−0.061,−0.003)	−0.034	0.057	(−0.146, 0.078)
$\zeta_{2}$	0.221	0.027	(0.168, 0.274)	0.252	0.076	(0.103, 0.401)
$\zeta_{3}$	0.314	0.029	(0.257, 0.370)	0.356	0.078	(0.203, 0.509)
$\zeta_{4}$	0.149	0.024	(0.102, 0.196)	0.194	0.084	(0.030, 0.359)
$\zeta_{5}$	0.265	0.024	(0.218, 0.312)	0.317	0.074	(0.172, 0.463)
ϕ	1.405			1.516	0.164	(1.195, 1.837)

**Table 3:** Estimation results with zero inflation: significance highlighting for 5% level (light green), 1% level (medium green), 0.1% level (dark green), and not significant at the 5% level (grey).

	Model	${\hat{\beta }}_{0}$	${\hat{\alpha }}_{1}$	${\hat{\alpha }}_{2}$	$\hat{\phi }$	$\hat{\omega }$	AIC
Anorexia	Pois-INAR(1)	2.215	0.338	-	-	0.669	183.00
		(0.428)	(0.074)	-	-	(0.069)	
	Pois-INAR(2)	2.692	0.249	0.118	-	0.752	179.85
		(0.536)	(0.090)	(0.070)	-	(0.061)	
	NB-INAR(1)	1.642	0.310	-	1.278	0.424	182.25
		(0.831)	(0.077)	-	(1.766)	(0.283)	
	NB-INAR(2)	2.363	0.225	0.120	2.668	0.630	179.94
		(0.794)	(0.093)	(0.073)	(3.561)	(0.178)	
Lesions	Pois-INAR(1)	2.042	0.175	-	-	0.372	276.26
		(0.278)	(0.071)	-	-	(0.077)	
	Pois-INAR(2)	2.055	0.110	0.176	-	0.464	271.32
		(0.333)	(0.073)	(0.073)	-	(0.089)	
	NB-INAR(1)	1.344	0.130	-	1.018	0.047	270.60
		(0.599)	(0.077)	-	(1.081)	(0.242)	
	NB-INAR(2)	1.252	0.084	0.166	0.920	0.103	267.76
		(0.754)	(0.076)	(0.075)	(1.218)	(0.313)	

## Data Availability

Data used in application 1 are provided by RKI [15] and published by NPGEO [16] at https://npgeo-corona-npgeo-de.hub.arcgis.com/datasets/dd4580c810204019a7b8eb3e0b329dd6_0/data (accessed on 16 March 2021). Data on animal submissions in application 2 is provided in the article by Aghababaei Jazi et al. [13]. The data set on corporate insolvencies is found in the supplementary material of Weiß and Feld [11]. Shapefiles for Germany used to create Figures in application 3 are provided by eurostat [27].

## Supplementary Materials

The following are available online at https://www.mdpi.com/article/10.3390/e23060666/s1, Julia and R code for all four applications.

## Appendix A

**Table A1:** Estimation results without zero inflation—significance highlighting for 5% level (light green), 1% level (medium green), 0.1% level (dark green), and not significant at the 5% level (gray).

	Model	${\hat{\beta }}_{0}$	${\hat{\alpha }}_{1}$	${\hat{\alpha }}_{2}$	$\hat{\phi }$	AIC
Anorexia	Pois-INAR(1)	0.511	0.385	-	-	225.05
		(0.087)	(0.073)	-	-	
	Pois-INAR(2)	0.450	0.353	0.098	-	224.70
		(0.088)	(0.078)	(0.072)	-	
	NB-INAR(1)	0.579	0.304	-	0.194	181.64
		(0.173)	(0.078)	-	(0.011)	
	NB-INAR(2)	0.545	0.220	0.118	0.139	180.37
		(0.188)	(0.096)	(0.078)	(0.059)	
Lesions	Pois-INAR(1)	1.172	0.173	-	-	294.77
		(0.146)	(0.068)	-	-	
	Pois-INAR(2)	0.976	0.145	0.132	-	292.17
		(0.153)	(0.068)	(0.067)	-	
	NB-INAR(1)	1.236	0.128	-	0.839	268.59
		(0.217)	(0.076)	-	(0.006)	
	NB-INAR(2)	1.017	0.084	0.164	0.608	265.85
		(0.214)	(0.076)	(0.075)	(0.248)	

**Table A2:** Model choice for each district.

District	Mean	Var	Model	Trend
Ahrweiler	3.11	3.39	Pois IID	No
Altenkirchen	3.03	5.34	NB-INGARCH(1, 1)	Yes
Alzey-Worms	2.76	4.61	NB-INGARCH(2, 1)	Yes
Bad Dürckheim	1.88	2.57	NB INARCH(2)	Yes
Bad Kreuznach	4.94	5.90	Pois IID	Partly
Bernkastell-Wittlich	3.25	4.58	Pois IID	Yes
Birkenfeld	2.29	3.22	NB-INARCH(1)	Yes
Cochem-Zell	1.29	1.72	NB IID	Partly
Donnersbergkreis	1.38	1.86	P-INGARCH(1, 1)	Partly
Eifelkr. Bitburg-Prüm	2.03	3.28	NB-IID	Partly
Frankenthal, kfr. S.	0.96	1.29	P-INGARCH(1, 1)	Partly
Gemersheim	1.71	1.93	Pois IID	No
Kaiserslautern, kfr. S.	2.95	4.59	P-INGARCH(2, 1)	Yes
Kaiserslautern	2.73	6.03	NB-INGARCH(2, 1)	Yes
Koblenz	3.49	4.79	P-INGARCH(1, 1)	Partly
Kusel	1.27	1.53	P-INARCH(2)	No
Landau i.d.P	0.83	0.91	Pois IID	No
Ludwigshafen	3.06	4.73	NB IID	Yes
Mainz	4.90	10.62	NB IID	Yes
Mainz-Bingen	4.55	8.21	P-INGARCH(2, 1)	Partly
Mayen-Koblenz	5.24	6.67	NB-INGARCH(1, 1)	Partly
Neustadt a.d.W.	1.06	1.23	P-INGARCH(1, 1)	Yes
Neuwied	6.73	11.84	P-INGARCH(2, 1)	Yes
Pirmasens	0.94	0.91	Pois IID	No
Rhein-Hunsrück-Kreis	2.56	3.07	Pois IID	Partly
Rhein-Lahn-Kreis	2.88	3.83	Pois IID	Partly
Rhein-Pfalz-Kreis	2.40	2.82	Pois IID	Yes
Speyer	1.01	1.04	Pois IID	Partly
Südliche Weinstraße	1.76	1.98	Pois IID	Yes
Südwestpfalz	1.61	1.87	Pois IID	Yes
Trier, kfr. S.	1.87	1.85	Pois IID	Yes
Trier-Saarburg	1.46	1.90	NB IID	No
Vulkaneifel	1.41	1.70	P-INGARCH(1, 1)	Yes
Westerwald	5.30	8.66	NB-INGARCH(1, 1)	Yes
Worms	2.94	8.88	NB-INGARCH(1, 1)	Yes
Zweibrücken	0.88	1.13	P-INARCH(1)	No

## References

[1] Alzaid A., Al-Osh M. (1988). First-Order Integer-Valued Autoregressive (INAR(1)) Process: Distributional and Regression Properties. Stat. Neerl..

[2] Ferland R., Latour A., Oraichi D. (2006). Integer-Valued GARCH Process. J. Time Ser. Anal..

[3] Bezanson J., Karpinski S., Shah V.B., Edelman A. (2012). Julia: A Fast Dynamic Language for Technical Computing. arXiv.

[4] Liboschik T., Fried R., Fokianos K., Probst P. tscount: Analysis of Count Time Series.

[5] Weiß C.H., Feld M.H.J.M., Mamode Khan N., Sunecher Y. (2019). INARMA Modeling of Count Time Series. Stats.

[6] Harte D. (2017). HiddenMarkov: Hidden Markov Models.

[7] Himmelmann L. HMM: HMM—Hidden Markov Models.

[8] Jackman S. pscl: Classes and Methods for R Developed in the Political Science Computational Laboratory; R Package Version 1.5.5.

[9] Zeileis A., Kleiber C., Jackman S. (2008). Regression Models for Count Data in R. J. Stat. Softw..

[10] Mouchet M. (2020). HMMBase—A Lightweight and Efficient Hidden Markov Model Abstraction.

[11] Weiß C.H., Feld M. (2019). On the performance of information criteria for model identification of count time Series. Stud. Nonlinear Dyn. Econom..

[12] Liboschik T., Kerschke P., Fokianos K., Fried R. (2016). Modelling interventions in INGARCH processes. Int. J. Comput. Math..

[13] Aghababaei Jazi M., Jones G., Lai C.D. (2012). First-order integer valued AR processes with zero inflated poisson innovations. J. Time Ser. Anal..

[14] Czado C., Gneiting T., Held L. (2009). Predictive Model Assessment for Count Data. Biometrics.

[15] RKI (2021). Robert-Koch-Institut: SurvStat@RKI 2.0. https://survstat.rki.de/.

[16] NPGEO (2021). RKI COVID19. https://npgeo-corona-npgeo-de.hub.arcgis.com/datasets/dd4580c810204019a7b8eb3e0b329dd6_0.

[17] World Health Organization (2020). Transmission of SARS-CoV-2: Implications for Infection Prevention Precautions. https://www.who.int/news-room/commentaries/detail/transmission-of-sars-cov-2-implications-for-infection-prevention-precautions.

[18] Christou V., Fokianos K. (2014). Quasi-Likelihood Inference for Negative Binomial Time Series Models. J. Time Ser. Anal..

[19] Mohammadpour M., Bakouch H., Shirozhan M. (2018). Poisson-Lindley INAR(1) model with applications. Braz. J. Probab. Stat..

[20] Schweer S., Weiß C.H. (2014). Compound Poisson INAR(1) processes: Stochastic properties and testing for overdispersion. Comput. Stat. Data Anal..

[21] Röhl K.H., Vogt G. (2019). Unternehmensinsolvenzen in Deutschland. https://www.iwkoeln.de/studien/iw-trends/beitrag/klaus-heiner-roehl-unternehmensinsolvenzen-in-deutschland-trendwende-voraus-449151.html.

[22] Li Q., Chen H., Zhu F. (2021). Robust Estimation for Poisson Integer-Valued GARCH Models Using a New Hybrid Loss. J. Syst. Sci. Complex..

[23] Xiong L., Zhu F. (2021). Minimum Density Power Divergence Estimator for Negative Binomial Integer-Valued GARCH Models. Commun. Math. Stat..

[24] Weiß C.H. (2021). Stationary count time series models. WIREs Comput. Stat..

[25] Möller T., Weiß C., Kim H.Y., Sirchenko A. (2018). Modeling Zero Inflation in Count Data Time Series with Bounded Support. Methodol. Comput. Appl. Probab..

[26] Quoreshi A.M.M.S. (2006). Bivariate Time Series Modeling of Financial Count Data. Commun. Stat. Theory Methods.

[27] Eurostat (2021). GISCO: Geographische Informationen und Karten. https://ec.europa.eu/eurostat/de/web/gisco/geodata/reference-data/administrative-units-statistical-units/nuts.

